# The Richmond Union Infirmary

**Published:** 1890-02-15

**Authors:** 


### The Hospital Workshop.

### XXI.—THE RICHMOND UNION INFIRMARY.

(By our Special Commissioner.)

A short time since some comment was made in the columns of The Hospital upon the nursing arrangements and accommodation at the above-mentioned institution. A full inquiry was at once courted by the Board of Guardians, and every facility for investigation extended to your Commissioner through the personal courtesy of the chairman, Colonel Sparks. An inspection in the company of that gentleman, together with the medical officer, the master of the house, and the present nurse, has enabled the writer to give the following account of the existing condition of affairs at the infirmary.

The average number of patients under treatment is about 130. Of these many suffer from chronic complaints, such as senile decay, paralysis, ulcers, phthisis, old-standing bronchitis, and cancers; while there are occasionally a few acute cases, pneumonias for example, but accidents, as a rule, are taken in at the local general hospital. These cases are distributed among a number of small wards, containing from eight to fourteen beds, and situated in various buildings that are scattered about in an awkward, haphazard, and inconvenient way. The main administrative block was originally a farmhouse, and some of the wards are strongly suggestive of converted farm buildings; but whatever the purpose for which they were erected in the first place, they could hardly be less suited to modern ideas of the requirements of pauper nursing. Of the wards, many open directly one into the other. In a female ward of thirteen beds, the windows were all shut, and ventilation appeared to depend on the open fireplace and some gratings in the ceiling. In this room are thirteen inmates of different ages, under charge of a pauper wardswoman, and there the thirteen women sleep, eat, and pass the day, that is to say, when the weather is not fine enough to admit of outdoor exercise. A neighbouring ward held ten patients, under the same conditions. The windows were all shut, and ventilation appeared to depend on one small grating in the wall, as there were no roof ventilators, valves, or ventilating bricks to be seen. Now, this state of affairs seems hardly creditable to the parish. Much of the evil—it may be at once stated—can be traced to an absolute want of structural plan in the buildings, a fact which, judging from the report to be afterwards quoted, is recognized by the committee.

Under present arrangements there is but one trained nurse for the whole of the patients. She is called a "day" nurse, but has to take night duty when necessary, and also attends the "lying in" cases, of which there were thirteen last year. The surgical dressing is done by this nurse-of-all-work. Half-a-dozen ulcers inspected at the time of our visit showed a clean and healthy appearance that plainly denoted careful attention. The nurse administers the medicines, which she herself fetches from the dispensary and keeps in a special cupboard outside each ward. There is practically no other skilled nursing. In each ward is an attendant chosen by the master from among the able-bodied paupers. Indeed, were the nurse to carry out personally the many duties that are nominally hers she would need to be a most exceptional woman in mind, body, and training. She is generally responsible for the state of the wards and of the bedding. Considering the nature of many of the cases, the statement that there are no bedsores amongst them reflects much credit upon the present occupier of the post. Hitherto there has been no regular night nurse, but the wards are walked through at intervals by a "sitter up," one for the men and one for the women. Should any emergency occur, the inmate in charge of the ward is supposed to report to the "sitter up," who crosses the yard to fetch the nurse. There is no way of showing that the "sitter up" has done his duty, and experience has proved beyond a doubt that, without some check, it is simply useless to rely upon rounds of the kind being made with any regularity. The "tell-tale" clock, which registers the exact moment of the attendant's visit, is the only reliable plan hitherto introduced for meeting the difficulty. As things are now managed at Richmond, it is obvious that in cases of urgency there might be considerable delay before help could be summoned. The ward attendant would have to wait for the uncertain visit of the "sitter up," who, in turn, would have to summon a nurse exhausted by the duties of the day. Besides, taking into consideration the number of cases under treatment, it is not altogether improbable that several emergencies might occur at one and the same time. The fact that anything of the sort has not hitherto happened would not be a sufficient argument against the need of a better provision for the future. At certain times, when danger is apprehended, an extra person is placed on duty for the purpose of waking up the ward attendant if necessary. Among any collection of sick people, however, the unforeseen is always to be expected. A case or two of sudden hæmorrhage, of fatal epilepsy, or of delirium might lead to consequences of an awkward nature. As a matter of fact, the nurse is not often called up at night. Her hours are from eight in the morning to ten at night, and it is natural that there should be a deal of reluctance in rousing her from what is doubtless a well-earned repose. When such a course is decided upon by the united wisdom of the ward-keeper and the "sitter-up," both unskilled people, be it remembered, the patient must indeed be in a bad state. It seems unwise, moreover, to throw the responsibility of waking up a tired official upon any unfortunate pauper.

The accommodation for the nurse has hitherto consisted of a small and inconvenient apartment in the central block. Lately, however, one of the old wards has been converted into a good-sized room, having windows rooms and a fireplace. By cutting off the sleeping portion with curtains better quarters will be provided than fall to the lot of many hospital nurses. An adjoining ward is being adapted in the same way for the assistant nurse shortly to be added to the staff, and telephonic communication will be carried to each of these rooms.

The children are placed in a large, new, and modern detached building. Although not on the sick list, it is proposed to place these children under the care of an assistant nurse, who will also take charge of a certain number of infirm or bed-ridden cases. This mixture of duties seems unadvisable. A specially-qualified nurse is required to look after helpless invalids, but hospital training is hardly needed to enable a woman to supervise healthy children. There are some good wards, at present empty, in this new building, which suggests the wing of a projected hospital that has not been carried out owing to a failure of funds or of energy. The addition of sufficient wards to accommodate all their patients would be a sound and radical step on the part of the Richmond Guardians. As already stated, a great part of the existing difficulties may be traced to the ill-arranged nature of the buildings, a fact which seems to be fully recognized in the report. Indeed, it is only fair to state that most of the defects now mentioned are pointed out in that document, and the appended copy will show that an effort has been made to face and to remedy them, with what ultimate success remains to be seen.

[Copy of Report.]

Your committee have attentively considered the question of substituting a staff of paid nurses for the system at present existing in the workhouse infirmary wards of employing pauper wardsmen and wardswomen under one paid nurse, and interrogated the workhouse medical officer, who expressed himself satisfied on the whole, but would be quite satisfied with the present system should a trained night nurse be appointed.

While fully recognizing that it is unwise to put paupers in posts of responsibility as well as the undoubted advantages of a system of trained and paid nurses where, as in most of the metropolitan and other large unions, the infirmaries are buildings specially constructed for the purpose and distinct from the workhouse, they are of opinion that it would be impossible to carry out such a system in the existing buildings of the Richmond workhouse, which are quite unsuited for the purpose, there being no accommodation available for a staff of paid nurses.

There are at least a dozen small sick wards, in each of which it is necessary that some attendant, paid or unpaid, shall be continually present.

There are very few cases of acute sickness, by far the greater number of those in the sick wards being cases of senile decay. Bedsores have for a considerable period been unknown, a proof that the present system cannot be very defective in its working.

Your committee beg to offer the following recommendations:—

1. That the sanction of the Local Government Board be obtained to the employment of a certificated night nurse at a salary of £25 per annum, rising annually £1 to £30. Also an assistant nurse to take charge of the children, give assistance in the infirmary wards as may be and to required, at a salary of £18 per annum, rising annually £1 to £22.

2. That the ward over the nursery be taken into use as a female infirmary ward for bed-ridden and chronic cases.

3. That the children's nurse be accommodated in the small room over the nursery, and the present nurse in No. 24, and the night nurse in No. 57 room.

4. That No. 61 room be kept for cases requiring isolation, and No. 56 room for an occasional sick ward.

5. That in cases of acute sickness the medical officer be empowered to obtain such temporary help as he may consider necessary from a nursing institution.

6. That telephonic communication be established between the rooms occupied by the nurses.

Dated 14th November, 1889.

Rob. W. Sparks. Jenny S. Foster Newton.

J. Whitcombe. Charlotte A. Glossop.

J. E. Hawksford. G. R. Cook.

R. F. Stopford.

With regard to this report a few remarks may be offered. The absence of proper accommodation for nurses cannot be accepted as a reason for not providing such a staff, if shown to be required in the interests of the patients. Because the paupers are badly housed it does not follow that they should be badly nursed. A definition of "proper accommodation" for paid nurses would be desirable as it presents itself to the mind of the medical officer. Speaking broadly, there is no very great difference between the treatment of paid and of unpaid nurses; and such invidious distinctions, where they exist, are hardly in accordance with the unselfish spirit that is inseparable from modern nursing. As to the statement that by far the greater number of cases are those of senile decay, it may be noted that out of nine cases in an overflow ward specially devoted to chronic cases, three were entered as ulcers, one paralysis, one "debility," one old age, one gout, one chronic bronchitis, and one a "cripple." Four out of fourteen were in bed, and three required surgical dressing. As to the "recommendations," it seems only reasonable that the permanent staff should be of sufficient strength to cope with all emergencies without relying on outside help, except, of course, in the event of any serious epidemic. The last item as it stands is obscure; probably the addition "and the wards" would correctly express the intention of the committee.

Rebuilding seems to be the necessary preliminary to the organization of a system of skilled nursing in this institution. It should be mentioned that the drainage arrangements appear to be good and modern. Indeed, the board seems to have every wish to do what is right. Future action, therefore, will mainly depend on the attitude of the ratepayers, who should afford substantial support to the guardians by moving them to procure a loan to enable them to rebuild and reconstruct to the extent necessary for the credit of Richmond. In any case the present state of affairs ought not to be tolerated longer, now public attention has brought them to light.

